# *Clostridium perfringens* α-Toxin Impairs Innate Immunity via Inhibition of Neutrophil Differentiation

**DOI:** 10.1038/srep28192

**Published:** 2016-06-16

**Authors:** Masaya Takehara, Teruhisa Takagishi, Soshi Seike, Kaori Ohtani, Keiko Kobayashi, Kazuaki Miyamoto, Tohru Shimizu, Masahiro Nagahama

**Affiliations:** 1 Department of Microbiology, Faculty of Pharmaceutical Sciences, Tokushima Bunri University, Yamashiro-cho, Tokushima 770-8514, Japan; 2 Department of Bacteriology, Graduate School of Medical Science, Kanazawa University, 13-1 Takara-Machi, Kanazawa, Ishikawa 920-8640, Japan; 3 Miyarisan Pharmaceutical Co., LTD, 1-10-3, Kaminakazato, Kita-ku, Tokyo 114-0016, Japan

## Abstract

Although granulopoiesis is accelerated to suppress bacteria during infection, some bacteria can still cause life-threatening infections, but the mechanism behind this remains unclear. In this study, we found that mature neutrophils in bone marrow cells (BMCs) were decreased in *C. perfringens*-infected mice and also after injection of virulence factor α-toxin. *C. perfringens* infection interfered with the replenishment of mature neutrophils in the peripheral circulation and the accumulation of neutrophils at *C. perfringens*-infected sites in an α-toxin-dependent manner. Measurements of bacterial colony-forming units in *C. perfringens*-infected muscle revealed that α-toxin inhibited a reduction in the load of *C. perfringens*. *In vitro* treatment of isolated BMCs with α-toxin (phospholipase C) revealed that α-toxin directly decreased mature neutrophils. α-Toxin did not influence the viability of isolated mature neutrophils, while simultaneous treatment of BMCs with granulocyte colony-stimulating factor attenuated the reduction of mature neutrophils by α-toxin. Together, our results illustrate that impairment of the innate immune system by the inhibition of neutrophil differentiation is crucial for the pathogenesis of *C. perfringens* to promote disease to a life-threatening infection, which provides new insight to understand how pathogenic bacteria evade the host immune system.

Neutrophils play an important role in the elimination of pathogenic bacteria by phagocytosis, killing and digesting them, which is the first line of defense of the innate immune system^1^,^2^,^3^. Normally, a certain number of neutrophils are sustained in a steady state through granulopoiesis, while the acceleration of granulopoiesis occurs during bacteremia to overwhelm the infection, which is termed emergency granulopoiesis^4^,^5^,^6^.

During infection with a gram-negative bacteria, endothelial cells in various organs sense lipopolysaccharide (LPS) from the bacteria through the MyD88-dependent Toll-like receptor 4 signaling pathway, leading to the activation of granulocyte colony-stimulating factor (G-CSF) release into the systemic circulation^7^. Endothelial cell-derived G-CSF acts on myeloid precursors, resulting in acceleration of the production of neutrophils in bone marrow and spleen^7^,^8^,^9^. Thus, the host innate immune system is precisely regulated to defeat pathogenic bacteria. Nevertheless, some bacteria cause neutropenia leading to serious and life-threatening infections in a clinical context. As a possible mechanism of neutropenia, blockage of myeloid differentiation during lethal sepsis due to *Pseudomonas aeruginosa* has been reported^10^. However, it is less well understood whether blockage of myeloid differentiation by bacterial toxins contributes to the progression of the infection, especially in the early phase of infection.

*Clostridium perfringens* is a gram-positive, anaerobic pathogenic bacterium^11^. Of five distinct subgroups, types A to E, *C. perfringens* type A causes gas gangrene in humans^12^. Gas gangrene caused by *C. perfringens* type A is accompanied by the destruction of muscle, shock, multiple organ failure, and death of patients^13^. *C. perfringens* infection progresses so rapidly that death precedes diagnosis in some patients, suggesting that *C. perfringens* can evade host innate immunity. Moreover, *C. perfringens* infection is characterized by an absence of polymorphonuclear leukocytes at the site of the infection^14^,^15^, but it has not been elucidated whether granulopoiesis is properly maintained during the infection.

Of the many toxins produced by *C. perfringens*, α-toxin is known to be a major virulence factor during infection and has two well-known enzyme activities: phospholipase C (PLC) and sphingomyelinase (SMase)^13^,^16^. In this study, to clarify whether the innate immune system is interfered with during *C. perfringens* type A infection, leading to rapid progression of the disease state, we evaluated granulopoiesis in *C. perfringens*-infected mice. This study demonstrated that mature neutrophils were decreased by infection in an α-toxin-dependent manner. Here, we demonstrate that *C. perfringens* α-toxin impairs innate immunity via the inhibition of neutrophil differentiation, which provides a new mechanism to explain how pathogenic bacteria evade the host immune system.

## Results

### Infection with *C. perfringens* diminishes mature neutrophils in bone marrow

In mouse bone marrow, granulocytes can be categorized into increasingly mature subsets by the expression of CD11b and Gr-1, as previously described^17^. Expression profiling of CD11b and Gr-1 in bone marrow cells (BMCs) from naive C57BL/6 mice identified three distinct populations defined as CD11b^+^Gr-1^high^, CD11b^+^Gr-1^low^, and CD11b^−^Gr-1^+^ (Fig. S1A). Giemsa staining of the sorted cells showed that the CD11b^+^Gr-1^high^ cell population contained mature neutrophils with segmented nuclei, the CD11b^+^Gr-1^low^ cell population represented an intermediate stage of maturation with incompletely condensed nuclei, and the CD11b^−^Gr-1^+^ cell population contained primitive precursor cells with a myeloblast-like morphology (Fig. S1A). Compared with the CD11b^+^Gr-1^high^ cell population, the CD11b^+^Gr-1^low^ and CD11b^−^Gr-1^+^ cell populations contained cells expressing a high level of cKit, a marker of immaturity^18^, which is consistent with the results of morphological analysis (Fig. S1B). Thus, the expression levels of CD11b and Gr-1 can be used to represent the stages of neutrophil maturation.

**Figure 1:**
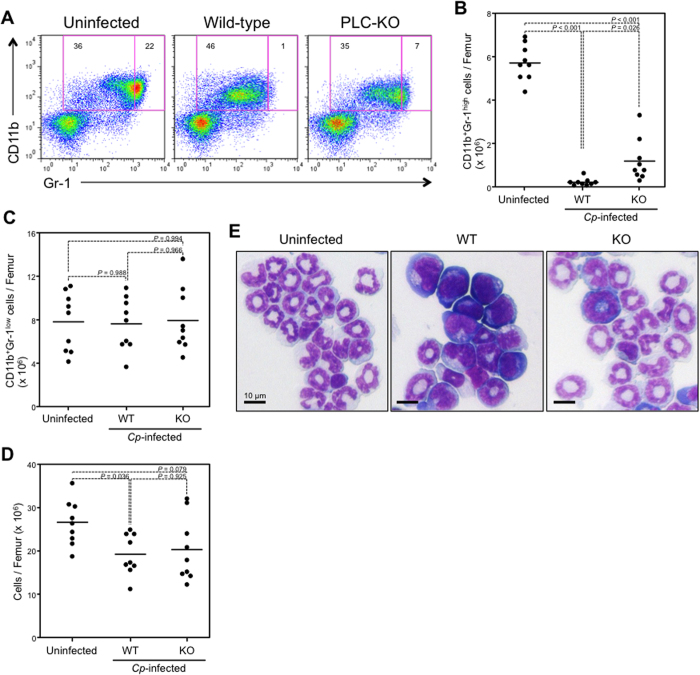
Mature neutrophils in bone marrow are decreased in *Clostridium perfringens*-infected mice. Mice were intramuscularly injected with 1 × 10^7^ colony-forming units (CFUs) of *C. perfringens* Strain 13 (WT, n = 9), PLC-KO (KO, n = 9), or TGY medium as a control (uninfected, n = 9), bone marrow cells (BMCs) were isolated from mice after 24 hours, and flow cytometry analysis was performed using a Guava easyCyte. Representative flow cytometry profile (**A**), the absolute number of CD11b^+^Gr-1^high^ mature neutrophils per femur (**B**), the absolute number of CD11b^+^Gr-1^low^ immature neutrophils per femur (**C**), and bone marrow cellularity (**D**) are shown. Magnetically isolated Gr-1^+^ cells from BMCs were stained with Giemsa (**E**). One-way analysis of variance was employed to assess statistical significance.

To investigate whether *C. perfringens* infection affects the maturation of neutrophils, BMCs were isolated from mice intramuscularly injected with *C. perfringens* type A, and flow cytometry analysis was performed. There were notable decreases in the proportion and number of CD11b^+^ Gr-1^high^ cells in *C. perfringens*-infected wild-type strain (Strain 13) mice compared with TGY medium-treated BMCs, whereas the decreases were attenuated in a *plc* gene-knockout mutant (PLC-KO) *C. perfringens*-infected mice (Fig. 1A,B). On the other hand, the number of CD11b^+^Gr-1^low^ cells was not affected by *C. perfringens* infection (Fig. 1C). Bone marrow cellularity was slightly decreased by infection, reflecting the loss of CD11b^+^Gr-1^high^ cells (Fig. 1D). Giemsa staining of isolated Gr-1^+^ cells from *C. perfringens*-infected BMCs showed that mature neutrophils with segmented nuclei had disappeared in the presence of α-toxin (Fig. 1E). These results indicated that *C. perfringens* infection preferentially decreases mature neutrophils in bone marrow.

**Figure 2:**
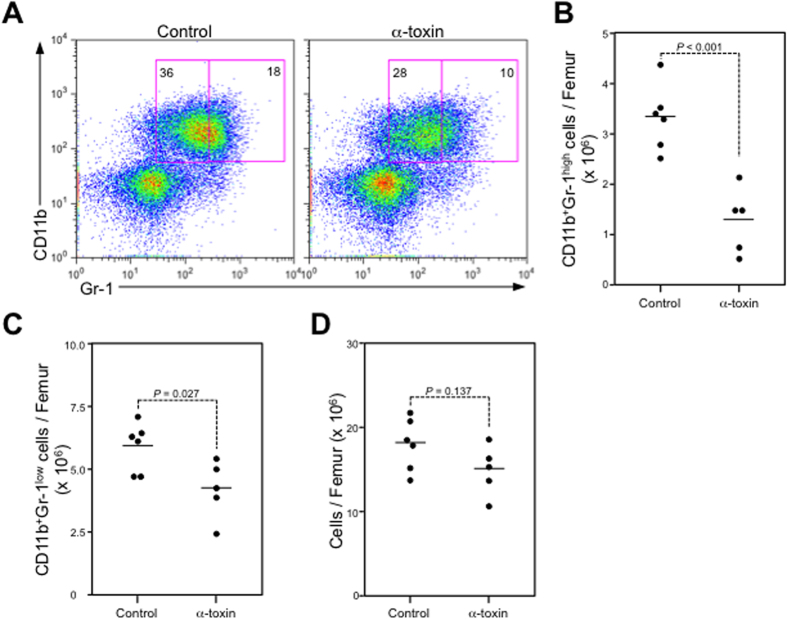
Mature neutrophils are decreased after injection with α-toxin. Mice were injected intravenously with 100 ng of purified α-toxin (α-toxin, n = 5) or Tris-buffered saline containing 0.25% gelatin as a control (control, n = 6), and flow cytometry analysis of BMCs isolated from the mice was performed with a Guava easyCyte. A representative flow cytometry profile is shown (**A**). The absolute number of CD11b^+^Gr-1^high^ cells (**B**), the absolute number of CD11b^+^Gr-1^low^ cells (**C**), and bone marrow cellularity (**D**) were determined. Two-tailed Student’s t-tests were used to assess statistical significance.

Next, we tested whether α-toxin was sufficient to induce a decrease in mature neutrophils in bone marrow. A single intravenous injection of purified α-toxin greatly decreased the proportion and number of CD11b^+^Gr-1^high^ cells, whereas those of CD11b^+^Gr-1^low^ cells were only slightly decreased in α-toxin-injected mice (Fig. 2A–C). Bone marrow cellularity was slightly decreased, but this was not significant (Fig. 2D). These results indicated that α-toxin plays an important role in the marked diminution of mature neutrophils by *C. perfringens* infection.

### α-Toxin interferes with the replenishment of mature neutrophils in the peripheral circulation

**Figure 3:**
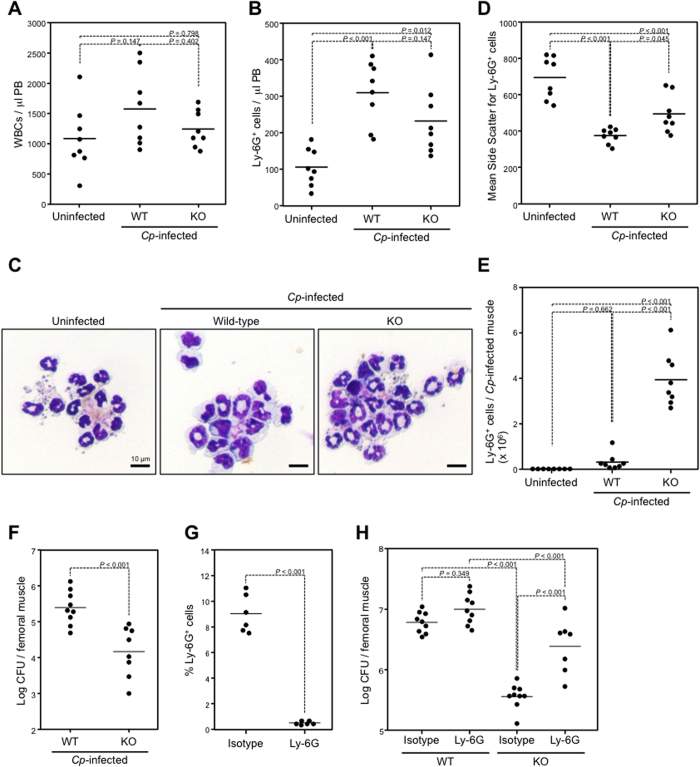
*Clostridium perfringens* infection interferes with replenishment of mature neutrophils in peripheral blood. Mice were injected intramuscularly with 1 × 10^7^ colony-forming units (CFUs) of *C. perfringens* Strain 13 (WT, n = 8–9), PLC-KO (KO, n = 8–9), or TGY medium as a control (uninfected, n = 8). (**A–D**) Peripheral white blood cells (WBCs) were isolated 24 hours after the infection. The number of WBCs was determined (**A**). WBCs were labeled, and the number of Ly-6G^+^ cells was determined (**B**). Magnetically isolated Ly-6G^+^ cells from WBCs were stained with Giemsa (**C**). Mean side-scatter intensity in the Ly-6G^+^ cell population is shown (**D**). (**E,F**) *C. perfringens*-infected femoral muscle was dissociated. The number of Ly-6G^+^ cells (**E**) and *C. perfringens* CFUs (**F**) in the suspension were determined. Mice were administrated intraperitoneally with an antibody against Ly-6G (Ly-6G) (**G,H**). The next day, the proportion of Ly-6G^+^ cells in peripheral WBCs was determined (**G**), and the mice were injected intramuscularly with 1 × 10^7^ CFUs of *C. perfringens* Strain 13 (WT) or PLC-KO (KO). *C. perfringens* CFUs in the muscle were determined 24 hours after infection (**H**). Rat IgG2a was used as an isotype control antibody (Isotype). One-way analysis of variance (**A,B,D,E,H**) or two-tailed Student’s t-test (**F,G**) were employed to assess statistical significance.

Next, we investigated whether *C. perfringens* infection influenced the replenishment of mature neutrophils in the peripheral circulation. Although the white blood cell (WBC) number in peripheral blood was not affected by *C. perfringens* infection, the number of Ly-6G^+^ neutrophils was increased compared with in uninfected mice (Fig. 3A,B). To assess the maturity of the neutrophils morphologically, we isolated peripheral Ly-6G^+^ cells from *C. perfringens*-infected mice and performed Giemsa staining. Figure 3C shows that the cells from uninfected control mice and PLC-KO-infected mice were mature neutrophils, whereas those from Strain 13-infected mice were immature cells with incompletely condensed nuclei. A comparison of side scatter intensity for Ly-6G^+^ cells, which is proportional to cell granularity, revealed that granulation was reduced by α-toxin producing *C. perfringens* infection (Fig. 3D). To evaluate the accumulation of neutrophils in *C. perfringens*-infected femoral muscle, we dissociated the muscle and determined the number of Ly-6G^+^ neutrophils. The numbers of neutrophils were similar in uninfected and Strain 13-infected muscles, whereas the cells dramatically increased in PLC-KO-infected muscle (Fig. 3E). Moreover, measurements of bacterial colony-forming units (CFUs) revealed that PLC-KO-infected mice were more efficient at reducing the load of *C. perfringens* compared with Strain 13-infected mice (Fig. 3F). By utilizing an antibody against Ly-6G, neutrophils can be specifically depleted *in vivo*^19^,^20^. To clarify that the accumulated neutrophils in PLC-KO-infected muscle contributed to the reduction in bacterial CFUs, mice were intraperitoneally administrated the antibody 1 day prior to *C. perfringens* infection. Figure 3G shows that administration of the antibody depleted Ly-6G^+^ neutrophils in peripheral blood. The depletion of neutrophils did not affect bacterial CFUs in Strain 13-infected muscle, whereas it greatly increased bacterial CFUs in PLC-KO-infected muscle, demonstrating that the accumulated neutrophils in PLC-KO-infected muscle were involved in the reduction of bacterial CFUs (Fig. 3H). Together, these results suggested that α-toxin interferes with the replenishment of mature neutrophils in the peripheral circulation, resulting in host innate immune deficiency.

### α-Toxin directly affects mature neutrophils *in vitro*

**Figure 4:**
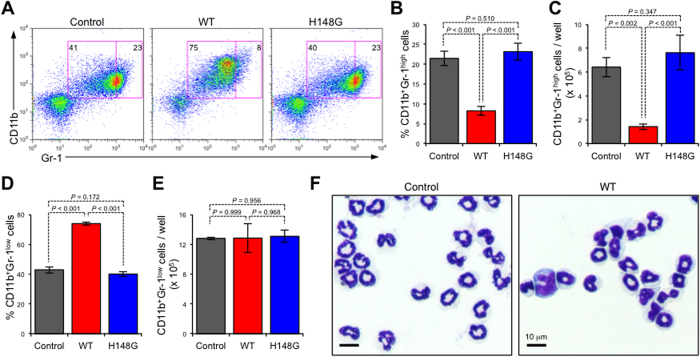
α-Toxin directly affects bone marrow cells. A total of 5 × 10^6^ bone marrow cells (BMCs; n = 3 per condition) were cultured for 24 hours in the presence or absence (control) of 100 ng/ml α-toxin (WT) or a variant α-toxin (H148G). Representative flow cytometry profile (**A**), the frequency and absolute number of CD11b^+^Gr-1^high^ neutrophils per culture well (**B,C**), and the frequency and absolute number of CD11b^+^Gr-1^low^ neutrophils per culture well (**D,E**) are shown. Magnetically isolated Gr-1^+^ cells from cultured BMCs were stained with Giemsa (**F**). Values are mean ± standard deviation. One-way analysis of variance was employed to assess statistical significance.

It has been reported that granulopoiesis could be ectopically affected by bacterial components during infection^7^. To test whether mature neutrophils were directly affected by α-toxin, we treated isolated BMCs with α-toxin *in vitro*. In α-toxin-treated BMCs, the proportion and number of CD11b^+^Gr-1^high^ cells were significantly decreased, whereas the proportion of CD11b^+^Gr-1^low^ cells was increased (Fig. 4A–D). Because the absolute numbers of CD11b^+^Gr-1^low^ cells were similar between α-toxin-treated and untreated conditions, the increase in the CD11b^+^Gr-1^low^ cell population in α-toxin-treated BMCs reflected the loss of CD11b^+^Gr-1^high^ cells (Fig. 4D,E). Previously, we reported that a variant α-toxin (H148G) lacked PLC and SMase activities^21^. The H148G variant α-toxin lost the ability to decrease the proportion and number of CD11b^+^Gr-1^high^ cells, demonstrating that α-toxin affects neutrophils in its enzyme activities-dependent manner (Fig. 4A–C). The disappearance of morphologically mature neutrophils in isolated Gr-1^+^ cells from α-toxin-treated BMCs was consistent with the results of the flow cytometry analysis (Fig. 4F). These results indicated that α-toxin directly influences BMCs to decrease the number of mature neutrophils.

**Figure 5:**
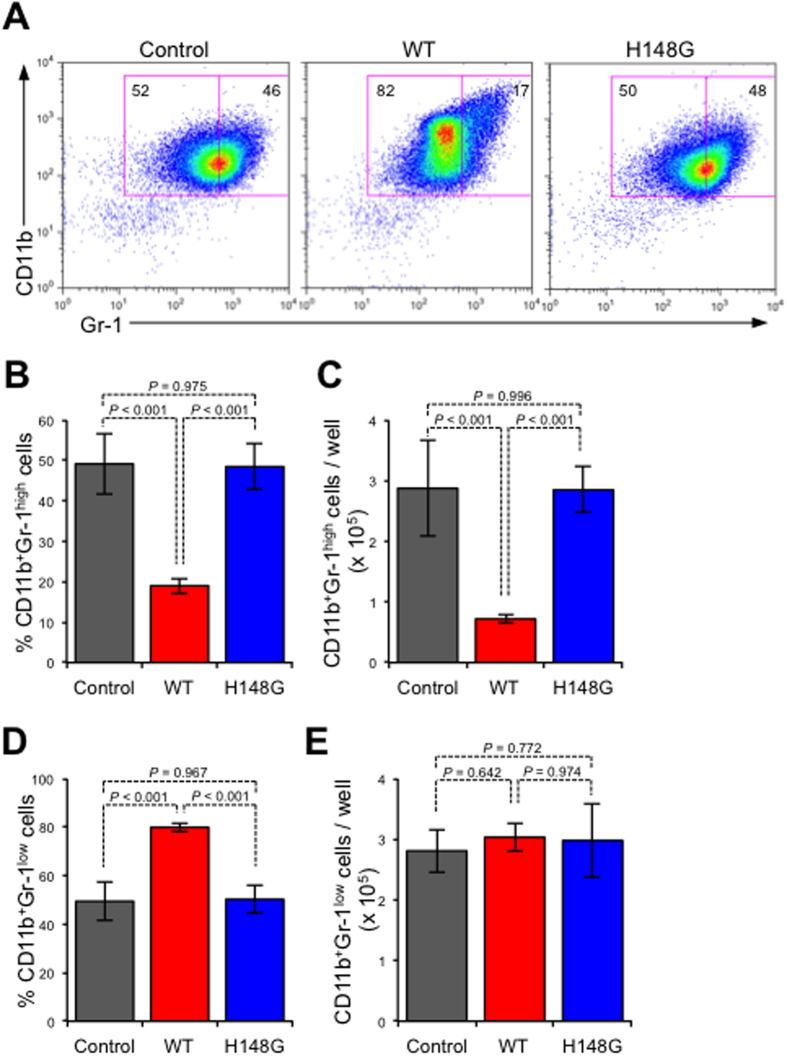
α-Toxin directly affects neutrophils. A total of 1 × 10^6^ magnetically isolated Gr-1^+^ cells (n = 6 per condition) were cultured for 24 hours in the presence or absence (Control) of 100 ng/ml α-toxin (WT) or a variant α-toxin (H148G). Representative flow cytometry profile (**A**), the frequency and absolute number of CD11b^+^Gr-1^high^ neutrophils per culture well (**B,C**), and the frequency and absolute number of CD11b^+^Gr-1^low^ neutrophils per culture well (**D,E**) are shown. Values are mean ± standard deviation. One-way analysis of variance was employed to assess statistical significance.

Next, we investigated the effect of α-toxin on isolated Gr-1^+^ cells. Almost all of the isolated Gr-1^+^ cells co-expressed CD11b, which means that the cells were CD11b^+^Gr-1^+^ cells (Fig. S2). When the CD11b^+^Gr-1^+^ cells were treated with α-toxin, the proportion and number of CD11b^+^Gr-1^high^ cells, but not CD11b^+^Gr-1^low^ cells, were decreased (Fig. 5). Also, the H148G variant α-toxin did not affect the proportion and number of CD11b^+^Gr-1^high^ cells.

To test whether α-toxin spreads to bone marrow and binds to neutrophils in *C. perfringens*-infected mice, we immunostained Gr-1^+^ cells isolated from mice with an antibody against α-toxin. Figure S3 shows that α-toxin was detected in cells from Strain 13-infected mice but not uninfected or PLC-KO-infected mice. Together, these results indicated that α-toxin directly affects neutrophils.

### α-Toxin inhibits neutrophil differentiation

**Figure 6:**
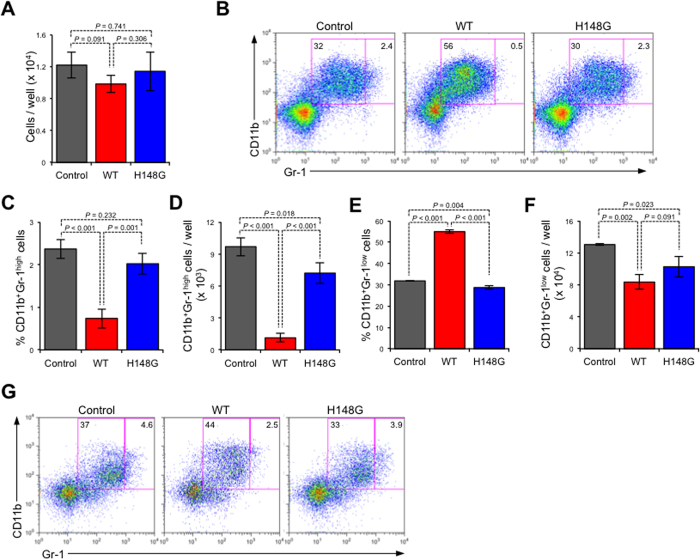
α-Toxin reduces the production but not cell viability of mature neutrophils *in vitro.* A total of 5 × 10^4^ sorted CD11b^+^Gr-1^high^ cells (**A**), n = 6 per condition), 7.5 × 10^5^ CD11b^+^Gr-1^high^-cell-depleted bone marrow cells (BMCs) (**B–F**), n = 3 per condition), or 1 × 10^5^ CD11b^−^Gr-1^+^ cells (**G**) were cultured for 24 hours in the presence or absence (Control) of 100 ng/ml α-toxin (WT) or a variant α-toxin (H148G). The number of viable cells per culture well was counted after trypan blue staining (**A**). Representative flow cytometry profile (**B,G**), the frequency and absolute number of CD11b^+^Gr-1^high^ neutrophils per culture well (**C,D**), and the frequency and absolute number of CD11b^+^Gr-1^low^ neutrophils per culture well (**E,F**) are shown. Freshly isolated BMCs were used as the gating reference (**B)**. Values are mean ± standard deviation. One-way analysis of variance was employed to assess statistical significance.

**Figure 7:**
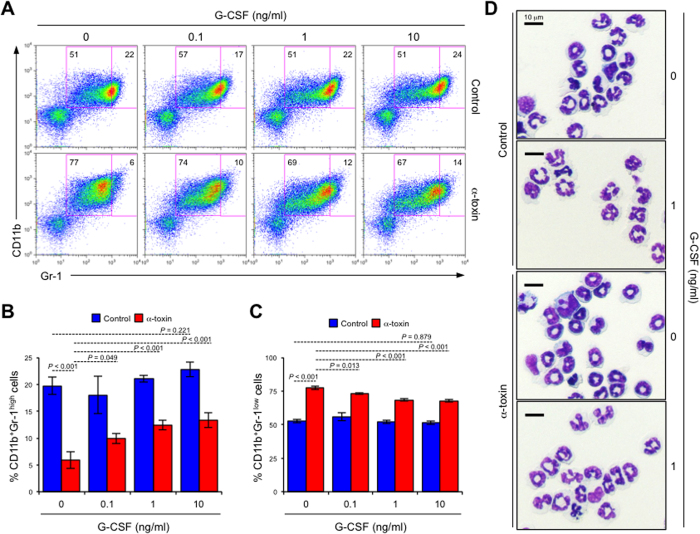
G-CSF induces differentiation of immature neutrophils treated with α-toxin. A total of 5 × 10^6^ BMCs (n = 4 per condition) were cultured for 24 hours in the presence or absence (control) of 100 ng/ml α-toxin (α-toxin) and the indicated concentration of purified mouse G-CSF. Representative flow cytometry profile (**A**), the frequencies of CD11b^+^Gr-1^high^ neutrophils (**B**) and CD11b^+^Gr-1^low^ neutrophils (**C**) are shown. Magnetically isolated Gr-1^+^ cells from cultured BMCs were stained with Giemsa (**D**). Values are mean ± standard deviation. One-way analysis of variance was employed to assess statistical significance.

At least two possible reasons for the α-toxin-induced decrease in mature neutrophils were suggested. One is that α-toxin specifically leads to cell death in CD11b^+^Gr-1^high^ cells, and the other is that it blocks the differentiation of immature neutrophils. We evaluated the number of viable cells in sorted CD11b^+^Gr-1^high^ cells after α-toxin treatment by counting and found that α-toxin did not influence the viability of the cells, showing that α-toxin has no apparent cytotoxicity with mature neutrophils (Fig. 6A). Next, we tested whether α-toxin blocked the production of CD11b^+^Gr-1^high^ cells using CD11b^+^Gr-1^high^ cell-depleted BMCs. As shown in Fig. S2, CD11b^+^Gr-1^high^ cells had been successfully removed from whole BMCs. After a 24-hour incubation of the cells, approximately 2.4% of CD11b^+^Gr-1^high^ cells emerged in the control medium, whereas cells treated with α-toxin contained less than 1% CD11b^+^Gr-1^high^ cells (Fig. 6B,C). The proportion and number of CD11b^+^Gr-1^high^ cells were not affected by treatment of the H148G variant α-toxin (Fig. 6B,C). The absolute number of CD11b^+^Gr-1^high^ cells decreased markedly in α-toxin-treated cells, but not in the H148G variant α-toxin-treated cells (Fig. 6D). On the other hand, α-toxin treatment induced an increase in the proportion of CD11b^+^Gr-1^low^ cells, while the absolute number of CD11b^+^Gr-1^low^ cells was decreased slightly (Fig. 6E,F). A similar result was obtained by using sorted CD11b^−^Gr-1^+^ cells (Fig. 6G). Furthermore, simultaneous treatment of BMCs with α-toxin and G-CSF, which is known to promote neutrophil differentiation^22^,^23^, attenuated the reduction in the proportion of CD11b^+^Gr-1^high^ cells or the increase in that of CD11b^+^Gr-1^low^ cells by α-toxin in a dose-dependent manner (Fig. 7A–C). Morphological analysis of the isolated Gr-1^+^ cells revealed that cells treated with α-toxin and G-CSF were more-fully differentiated than those treated with α-toxin alone (Fig. 7D). These results demonstrated that G-CSF induces the differentiation of immature neutrophils treated with α-toxin. Together, our results strongly suggested that blockage of neutrophil differentiation is involved in the reduction of mature neutrophils by α-toxin. Because the H148G variant α-toxin lost the ability to inhibit the differentiation of neutrophils, enzyme activities are necessary for α-toxin to impair neutrophil differentiation.

## Discussion

The clinical situation in *C. perfringens*-infected patients is complex. Various pathogenic bacteria are occasionally identified in *C. perfringens*-infected patients, meaning that polymicrobial infection is likely to occur clinically^24^. In addition, *C. perfringens* infection is characterized by an absence of polymorphonuclear leukocytes at the site of the infection^14^,^15^. In this study, we found that *C. perfringens* infection reduced mature neutrophils in bone marrow, peripheral blood, and *C. perfringens*-infected muscle in an α-toxin-dependent manner. These results suggested that α-toxin impairs granulopoiesis and interferes with the replenishment of mature neutrophils in the peripheral circulation leading to reduced recruitment of neutrophils to the *C. perfringens* infection site. Thus, *C. perfringens* infection impairs the host immune system via the systemic reduction of mature neutrophils, which could explain polymicrobial infection in patients and the absence of polymorphonuclear leukocytes at the site of infection.

*C. perfringens* infection markedly reduced the number of mature neutrophils in bone marrow, while a *plc* gene-knockout only partially attenuated the reduction. These results suggested that some other bacterial components also contribute to this phenomenon. Perfringolysin O, a cholestrerol-dependent cytolysin, is known as a major toxin produced by *C. perfringens* type A strains^25^,^26^. Purified perfringolysin O has been shown to be cytotoxic to polymorphonuclear leukocytes and macrophages^27^,^28^,^29^. *C. perfringens* is also known to produce other toxins and enzymes including a collagenase, hyaluronicdase, sialidases and the cysteine protease α-clostripain^25^,^30^. In the present study, we have not tested whether these bacterial components affect the number of mature neutrophils in bone marrow. Therefore, the possibility cannot be excluded that not only α-toxin but also the other bacterial components affect production or cell viability of neutrophils in *C. perfringens*-infected mice.

*C. perfringens* infection slightly increased the number of Ly-6G^+^ neutrophils in peripheral blood, but morphological analysis of the Ly-6G^+^ cells revealed that the increased neutrophils were immature cells with incompletely condensed nuclei. Also, a comparison of side scatter intensity for Ly-6G^+^ cells revealed that granulation was reduced in *C. perfringens*-infected peripheral neutrophils, and a *plc* gene-knockout slightly attenuated the reduction. Notably, the numbers of infiltrated neutrophils were similar in uninfected and Strain 13-infected muscles, whereas the cells dramatically increased in PLC-KO-infected muscle, demonstrating that the infiltration of neutrophils into *C. perfringens*-infected muscle was almost completely inhibited by α-toxin. These results suggested that α-toxin-induced impairment of neutrophil maturation might be insufficient to account for the dramatic inability of the cells to migrate to the infected sites. It has been reported that α-toxin mediates formation of platelet-leukocyte aggregates leading to vascular occlusion and induces a marked reduction in microvascular perfusion^31^,^32^. The platelet-leukocyte aggregates were also reported to impede neutrophil extravasation^33^. Therefore, the possibility cannot be excluded that the dramatic reduction of infiltrated neutrophils into *C. perfringens*-infected muscle is due not only to the impairment of neutrophil maturation, but also to reductions in microvascular perfusion and neutrophil extravasation.

In the present study, we demonstrated that the absolute number of CD11b^+^Gr-1^high^ cells was greatly decreased by α-toxin *in vitro*, whereas α-toxin did not influence the viability of the cells. Stevens *et al*. reported that α-toxin had no apparent toxic effect on human polymorphonuclear leukocytes^27^. A possible explanation for the decrease in mature neutrophils by α-toxin with no direct cytotoxicity is that the half-life of neutrophils is very short. As shown in Fig. 6A, a total of 5 × 10^4^ sorted CD11b^+^Gr-1^high^ cells were cultured in the experiment, and only around 25% of the cells were still alive after 24 hours in control medium, suggesting that the half-life of mature neutrophils is much less than 24 hours. Basu *et al*. reported that the half-life of peripheral neutrophils was 11.4 hours in mouse blood^34^, which is consistent with our result. These results suggested that a certain number of mature neutrophils are sustained through granulopoiesis *in vivo* and *in vitro*. Therefore, the blockage of neutrophil differentiation by α-toxin can cause a decrease in the absolute number of mature neutrophils without cytotoxicity. The reduction in mature neutrophils in bone marrow by *C. perfringens* infection could be explained by the same reason. Together, continuous granulopoiesis is necessary to sustain a certain number of mature neutrophils during bacterial infection, and the findings in this study suggested that the blockage of differentiation leads to a reduction in cells numbers in a short period of time.

**Figure 8:**
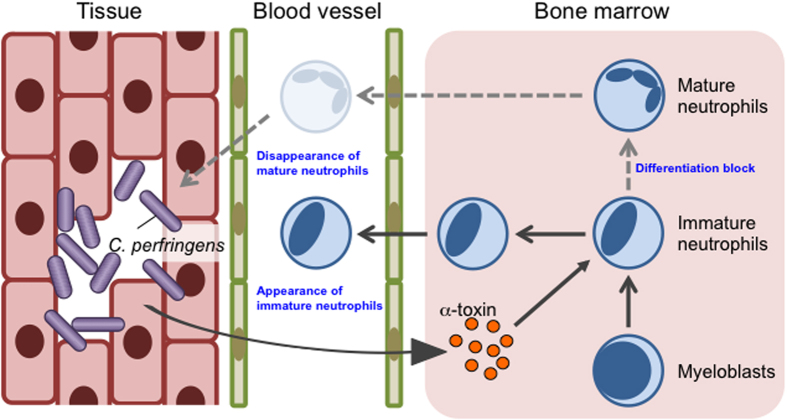
Model of impaired innate immunity by inhibition of granulopoiesis in a *Clostridium perfringens*-infected host. Infection with *C. perfringens* diminishes mature neutrophils in bone marrow leading to impairment of replenishment of mature neutrophils in the peripheral circulation, resulting in host innate immune deficiency. The major virulence factor of *C. perfringens*, α-toxin, plays an important role in this phenomenon by directly blocking neutrophil differentiation. Together, impairment of the innate immune system by the inhibition of granulopoiesis is crucial for the pathogenesis of *C. perfringens*.

A growing body of scientific evidence has indicated that the host innate immune system is precisely regulated via the activation of granulopoiesis in the fight against infection^4^; however, the mechanisms by which some bacteria overwhelm the immune system to cause serious and life-threatening infection are still poorly understood. Here, we demonstrated that *C. perfringens* type A infection impaired neutrophil differentiation to cause a reduction in mature neutrophils in bone marrow in an α-toxin dependent manner. Moreover, we found that α-toxin interfered with the replenishment of mature neutrophils in the peripheral circulation accompanied by decreased efficiency at reducing the load of *C. perfringens* in infected muscle, suggesting that the innate immune system is impaired by α-toxin. Together, our results illustrate that the impairment of the innate immune system by the inhibition of granulopoiesis is crucial for the pathogenesis of *C. perfringens* to promote disease to a life-threatening infection (Fig. 8). We hope that our results provide new insight to understand how pathogenic bacteria evade the host immune system.

## Methods

### Mice

C57BL/6J mice were purchased from Charles River Laboratories Japan, Inc. The mice were kept in a specific pathogen-free animal facility at Tokushima Bunri University. For all experiments, mice aged more than 8 weeks old were used. Animal experiments were approved by the Animal Care and Use Committee of Tokushima Bunri University, and procedures were performed in accordance with institutional guidelines (approval numbers: 14-2 and 15-3). The institutional guidelines conform to the Fundamental Guidelines for Proper Conduct of Animal Experiment and Related Activities in Academic Research Institutions under the jurisdiction of the Ministry of Education, Culture, Sports, Science and Technology, 2006.

### Reagents and strains

Fluorescein isothiocyanate (FITC)-, phycoerythrin (PE)-, or allophycocyanin (APC)-conjugated specific antibodies against mouse CD11b (clone M1/70), Ly-6G/6C (Gr-1, clone RB6-8C5), Ly-6G (clone 1A8), or CD117 (cKit, clone 2B8), and purified rat anti-mouse CD16/CD32 (Fc Block) were purchased from BD Biosciences. A specific antibody against *C. perfringens* α-toxin was prepared as described previously^35^. Giemsa’s azur eosin methylene blue solution was purchased from Merck. Mouse G-CSF was from Miltenyi Biotec. All other chemicals were of the highest grade available from commercial sources. *C. perfringens* wild-type Strain 13 and *Bacillus subtilis* ISW1214 were used in this study.

### Construction of mutant strain

The *Eco*RI-*Hin*dIII fragment containing the plc gene was cloned into pUC19 (pOT01). A 2.1 kb *Bam*HI-*Bgl*II fragment containing the tetracycline resistance gene was inserted into the *Bam*HI site of pOT01 located in the internal region of the *plc* gene^36^. The resultant plasmid, pOT11, was used to transform *C. perfringens* Strain 13. Transformants resulting from homologous recombination were screened on blood agar plates containing 2.5 μg/ml tetracycline, and α-toxin negative colonies were picked up by checking hemolysis. Allelic-exchange mutation of the *plc* gene due to a double cross-over recombination was confirmed by Southern blotting. Then, transformants were cultured on egg-yolk agar plates and lecithinase activity was checked.

### Purification of wild-type and variant α-toxin

Purification of wild-type or H148G variant α-toxin was performed as described previously^21^,^37^. Briefly, recombinant forms of pHY300PLK harboring the structural genes of wild-type or H148G variant α-toxin were introduced into *B. subtilis* ISW1214 by transformation, and the transformants were cultured in Luria-Bertani broth containing 50 μg/ml ampicillin with continuous aeration. Wild-type or H148G variant α-toxin secreted into the culture medium was purified chromatographically.

### Bone marrow cell isolation and culture

BMCs were isolated by crushing femurs and tibias in phosphate-buffered saline (PBS) supplemented with 2% heat-inactivated fetal bovine serum (FBS; AusGeneX), and filtered through a 40 μm mesh. Red blood cells were hemolyzed with lysis buffer (ACK lysing buffer, GIBCO). The number of living cells was counted after trypan blue staining. Isolated BMCs were cultured at 37 °C in RPMI 1640 medium supplemented with 10% FBS, 100 units/ml penicillin, and 100 μg/ml streptomycin.

### Bacterial culture and infection

*C. perfringens* Strain 13 or PLC-KO was grown in TGY medium in anaerobic conditions at 37 °C. Exponentially growing bacteria were harvested, washed, re-suspended in TGY medium, and injected into the left femoral muscle of mice. To quantify CFUs, residual bacteria were serially diluted, plated on BHI agar plates, and cultured anaerobically at 37 °C. BMCs were isolated from the right femur of the mice 24 hours after injection.

### Dissociation of *C. perfringens*-infected femoral muscle

*C. perfringens*-infected femoral muscle was isolated 24 hours after the infection. To quantify *C. perfringens* CFUs, isolated muscle was cut into small pieces of 2–4 mm in TGY medium and dissociated in a gentleMACS C tube (Miltenyi Biotec) using a gentle MACS dissociator (Miltenyi Biotec). The supernatant was serially diluted, plated on BHI agar plates, and cultured anaerobically at 37 °C.

To determine the number of Ly-6G^+^ cells in the infected muscle, isolated muscle was dissociated using Skeletal Muscle Dissociation Kit (Miltenyi Biotec) in accordance with the manufacturer’s protocol. Briefly, the muscle was cut into small pieces in Dulbecco’s modified Eagle’s medium (DMEM) medium containing Enzyme A, D, and P, and dissociated using a gentle MACS dissociator. The cell suspension was filtered through a 40 μm mesh after red blood cells were hemolyzed with lysis buffer. Flow cytometry analysis was performed as described below.

### Neutrophil depletion

Depletion of neutrophils in mice was performed as described previously^19^. An antibody (500 μg) against mouse Ly-6G (clone 1A8) (Bio X Cell) was administered intraperitoneally into mice prior to infection with *C. perfringens*. Rat IgG2a (clone 2A3) (Bio X Cell) was used as an isotype control antibody.

### Flow cytometry analysis

Antibodies described in the Reagents and Strains section were diluted with PBS containing 2% FBS and used to label cells after blocking Fc-receptors with purified rat anti-mouse CD16/CD32. The labeled cells were analyzed or sorted using a FACS Aria II (BD Biosciences) or a Guava easyCyte (Millipore). Data were analyzed using FlowJo (Tree Star) software.

### Magnetic cell isolation

Gr-1^+^ or Ly-6G^+^ cell isolation were performed using an EasySep system (StemCell Technologies) in accordance with the manufacturer’s protocol, with some modifications. In brief, cells were labeled with PE-conjugated specific antibodies against Gr-1 or Ly-6G. Antibody conjugation to magnetic nanoparticles was achieved through incubation with EasySep PE Selection cocktail followed by additional incubation with EasySep Magnetic Nanoparticles. The cells were separated using EasySep Magnet (StemCell Technologies). To deplete CD11b^+^Gr-1^high^ cells from BMCs, the sequential separation procedure was repeated twice.

### Immunofluorescence microscopy

Isolated Gr-1^+^ cells were cytospinned onto microscopic glass slides, fixed with 4% paraformaldehyde in PBS at room temperature for 15 min, and blocked with Blocking One Histo (Nacalai Tesque, Inc.). The samples were then incubated with a primary antibody against α-toxin. After washing, samples were incubated with the secondary antibody conjugated with Alexa Fluor 488 (Molecular Probes). Nuclei were stained with 4′,6-diamino-2-phenylindole (DAPI). Images were captured on a confocal laser-scanning fluorescence microscope (Nikon A1, Nikon instruments).

### Statistical analysis

All statistical analyses were performed with Easy R (Saitama Medical Center, Jichi Medical University)^38^. Differences between two groups were evaluated using two-tailed Student’s t-test. One-way analysis of variance (ANOVA) followed by the Tukey test was used to evaluate differences among three or more groups. Differences were considered to be significant for values of P < 0.05.

## Additional Information

**How to cite this article**: Takehara, M. *et al*. *Clostridium perfringens* α-Toxin Impairs Innate Immunity via Inhibition of Neutrophil Differentiation. *Sci. Rep.* **6**, 28192; doi: 10.1038/srep28192 (2016).

## Supplementary Material

Supplementary Information
