# Supplementary material for: Clostridium perfringens α-Toxin Impairs Innate Immunity via Inhibition of Neutrophil Differentiation
Source: Sci Rep. 2016 Jun 16;6:28192. doi: 10.1038/srep28192 (PMC4910053; doi:10.1038/srep28192)
Supplement: Supplementary Information [file srep28192-s1.pdf]

Clostridium perfringens α-Toxin Impairs Innate Immunity via Inhibition of Neutrophil Differentiation

Masaya Takehara[1,*], Teruhisa Takagishi[1], Soshi Seike[1], Kaori Ohtani[2,3], Keiko Kobayashi[1], Kazuaki Miyamoto[1], Tohru Shimizu[2], Masahiro Nagahama[1,*]

[1]Department of Microbiology, Faculty of Pharmaceutical Sciences, Tokushima Bunri University, Yamashiro-cho, Tokushima 770-8514, Japan

[2]Department of Bacteriology, Graduate School of Medical Science, Kanazawa University, 13-1 Takara-Machi, Kanazawa, Ishikawa 920-8640, Japan

[3]Miyarisan Pharmaceutical Co., LTD, 1-10-3, Kaminakazato, Kita-ku, Tokyo 114-0016, Japan

[*]Corresponding authors

E-mail: mtakehara@ph.bunri-u.ac.jp (MT), and nagahama@ph.bunri-u.ac.jp (MN)

Table of Contents

Supplementary Figures S1-S3

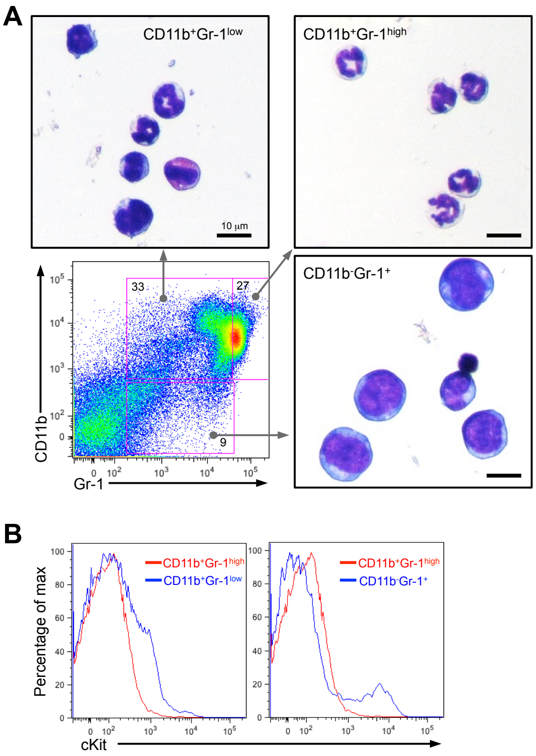

Figure S1. Expression of CD11b and Gr-1 represents the stages of neutrophil maturation. Bone marrow cells (BMCs) from C57BL/6 mice were labeled with specific antibodies against CD11b, Gr-1, and cKit. The cells were analyzed or sorted using a FACS Aria II. Three distinct populations were identified, and Giemsa staining of sorted CD11b^{+}Gr-1^{high}, CD11b^{+}Gr-1^{low}, and CD11b^{-}Gr-1^{+} cells was performed (A). The expression of cKit in the CD11b^{+}Gr-1^{low} and CD11b^{-}Gr-1^{+} cell populations (blue) was compared with that of the CD11b^{+}Gr-1^{high} cell population (red) (B).

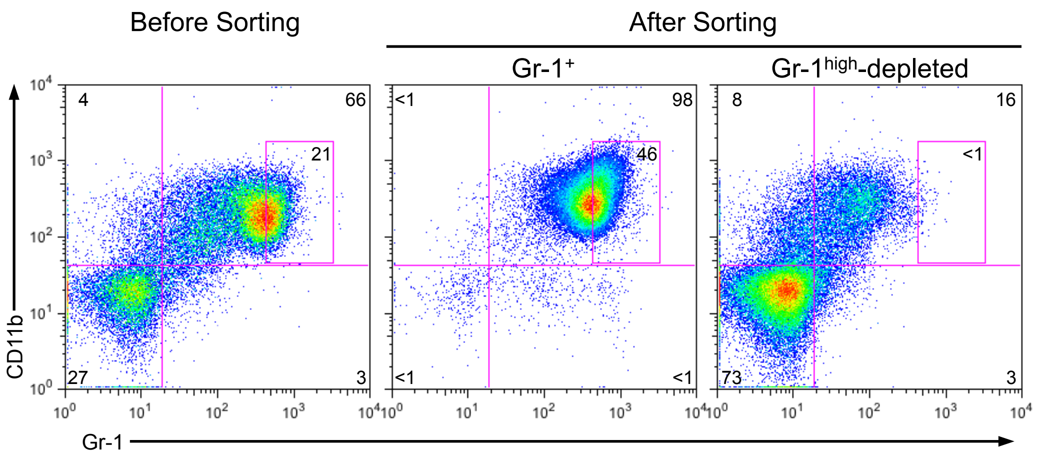

Figure S2. Preparation of Gr-1$^+$ cells and CD11b$^+$Gr-1^{high} cell-depleted bone marrow cells. Preparation of Gr-1$^+$ cells (Gr-1$^+$) and CD11b$^+$Gr-1^{high} cell-depleted Bone marrow cells (BMCs) (Gr-1^{high}-depleted) was performed using the EasySep system, as described in the Materials and Methods. Isolated cells were labeled with a specific antibody against CD11b, and flow cytometry analysis was performed using a Guava easyCyte. Almost all of the isolated Gr-1$^+$ cells co-expressed CD11b, which means that the isolated cells were CD11b$^+$Gr-1$^+$ cells. CD11b$^+$Gr-1^{high} cell-depleted BMCs contained less than 1% of CD11b$^+$Gr-1^{high} cells.

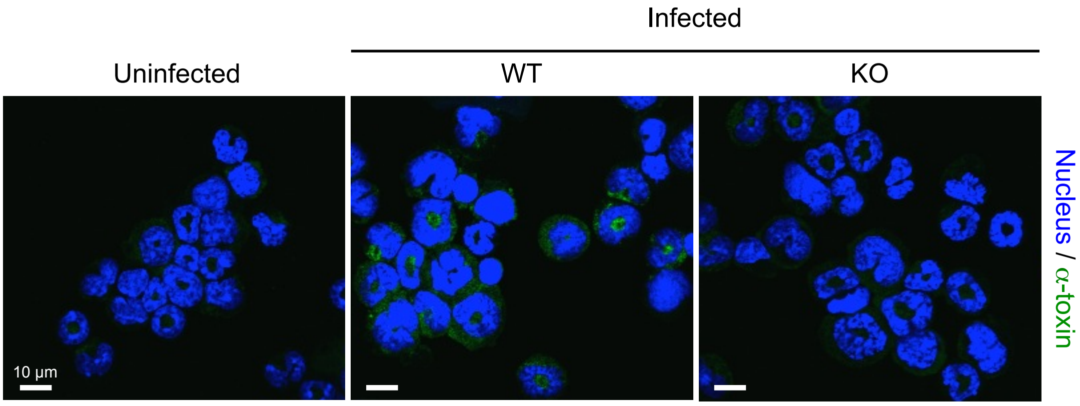

Figure S3. α-Toxin binds to bone marrow neutrophils in *Clostridium perfringens*-infected mice. Mice were intramuscularly injected with 1 × 10^7 CFU of *C. perfringens* Strain 13 (WT), PLC-KO (KO), or TGY medium as a control (uninfected), and bone marrow cells (BMCs) were isolated from the mice after 24 hours. Magnetically isolated Gr-1^+ cells from BMCs were incubated with an antibody against α-toxin. After incubation with a secondary antibody, the cells were inspected by confocal laser scanning microscopy.
